# High serum uric acid trajectories are associated with risk of myocardial infarction and all-cause mortality in general Chinese population

**DOI:** 10.1186/s13075-022-02812-y

**Published:** 2022-06-21

**Authors:** Xue Tian, Yingting Zuo, Shuohua Chen, Shouling Wu, Anxin Wang, Yanxia Luo

**Affiliations:** 1 Department of Epidemiology and Health Statistics, School of Public Health, Capital Medical University, No.10 Xitoutiao, You’anmen Wai, Fengtai District, Beijing, 100069 China; 2 Beijing Municipal Key Laboratory of Clinical Epidemiology, Beijing, China; 3 Department of Cardiology, Kailuan Hospital, North China University of Science and Technology, 57 Xinhua East Rd, Tangshan, 063000 China; 4 China National Clinical Research Center for Neurological Diseases, Beijing Tiantan Hospital, Capital Medical University, No.119 South 4th Ring West Road, Fengtai District, Beijing, 100070 China; 5 Department of Neurology, Beijing Tiantan Hospital, Capital Medical University, Beijing, China

**Keywords:** Serum uric acid, Trajectories, Myocardial infarction, All-cause mortality

## Abstract

**Background:**

Long-term patterns of serum uric acid (SUA) and their association with the risk of myocardial infarction (MI) and mortality are poorly characterized as prior studies measured SUA at a single time point. This study aimed to identify SUA trajectories and determine their associations with incident MI and all-cause mortality.

**Methods:**

We included 85,503 participants who were free of MI in or prior 2012 from the Kailuan study. SUA trajectories during 2006–2012 were identified by group-based trajectory modeling. Cox proportional hazard models were used to assess the association of SUA trajectories with MI and all-cause mortality.

**Results:**

We identified three SUA trajectories during 2006–2012: low-stable (*n*=44,124, mean SUA: 236–249 μmol/L), moderate-stable (*n*=34,431, mean SUA: 324–354 μmol/L) and high-stable (*n*=6,984, mean SUA: 425–463 μmol/L). During a median follow-up of 6.8 years, we documented 817 (0.96%) incident MI and 6498 (7.60%) mortality. Compared with the low-stable group, high-stable group experienced a higher risk of MI (hazard ratio [HR], 1.35; 95% confidence [CI], 1.07–1.71) and all-cause mortality (HR, 1.22; 95% CI, 1.12–1.33). Multiple sensitivity analyses yielded similar results. Additionally, the association of SUA trajectory with MI and all-cause mortality was more pronounced in individuals without a history of hypertension (*P*-interaction=0.0359) and those aged <60 years (*P*-interaction<0.0001), respectively.

**Conclusions:**

Higher SUA trajectories were associated with altered risk of MI and all-cause mortality, suggesting that monitoring SUA trajectory may assist in identifying subpopulations at higher risk of MI and all-cause mortality.

**Supplementary Information:**

The online version contains supplementary material available at 10.1186/s13075-022-02812-y.

## Introduction

Serum uric acid (SUA) is the end-product of purine metabolism via xanthine oxidoreductase in the human body and is mainly eliminated by the kidney and the intestinal tract [1]. SUA has been shown to have antioxidant and pro-inflammatory properties [2–4]; elevated SUA is associated with a wide variety of adverse health outcomes, such as hypertension [5], diabetes [6], and chronic kidney disease [7]. However, whether SUA is associated with higher risk of myocardial infarction (MI) and mortality has long been under debate [8–17]. Evidence from previous epidemiological studies yield inconsistent findings and was mainly limited by a single measurement of SUA. Thus, current evidence on the association of SUA with MI and mortality needs to be interpreted with caution, considering the wide heterogeneity in study designs and methodological limitations.

Since SUA changes over time, several previous studies focused on the effect of changes in SUA on the risk of cardiovascular disease and all-cause mortality, whereas the conclusions were controversial. However, changes in SUA in the aforementioned studies were defined on the basis of 2 measurements of SUA levels and were unable to account for the potential heterogeneity in changes in SUA levels. In addition, the study subjects were mainly patients with certain diseases, which further limits the generalizability of their results to the general population. To date, long-term patterns in SUA and their association with incident MI and all-cause mortality in the general population are still poorly characterized. Because high SUA is potentially one of the modifiable mediators of cardiovascular diseases and mortality, effective treatment and prevention interventions should have a profound favorable impact on public health [18].

Therefore, the aims of this study were to identify SUA trajectories over a 6-year exposure period and to examine the association between SUA trajectories and subsequent risk of MI and all-cause mortality in Chinese general population.

## Methods

### Study population

**Fig. 1:**
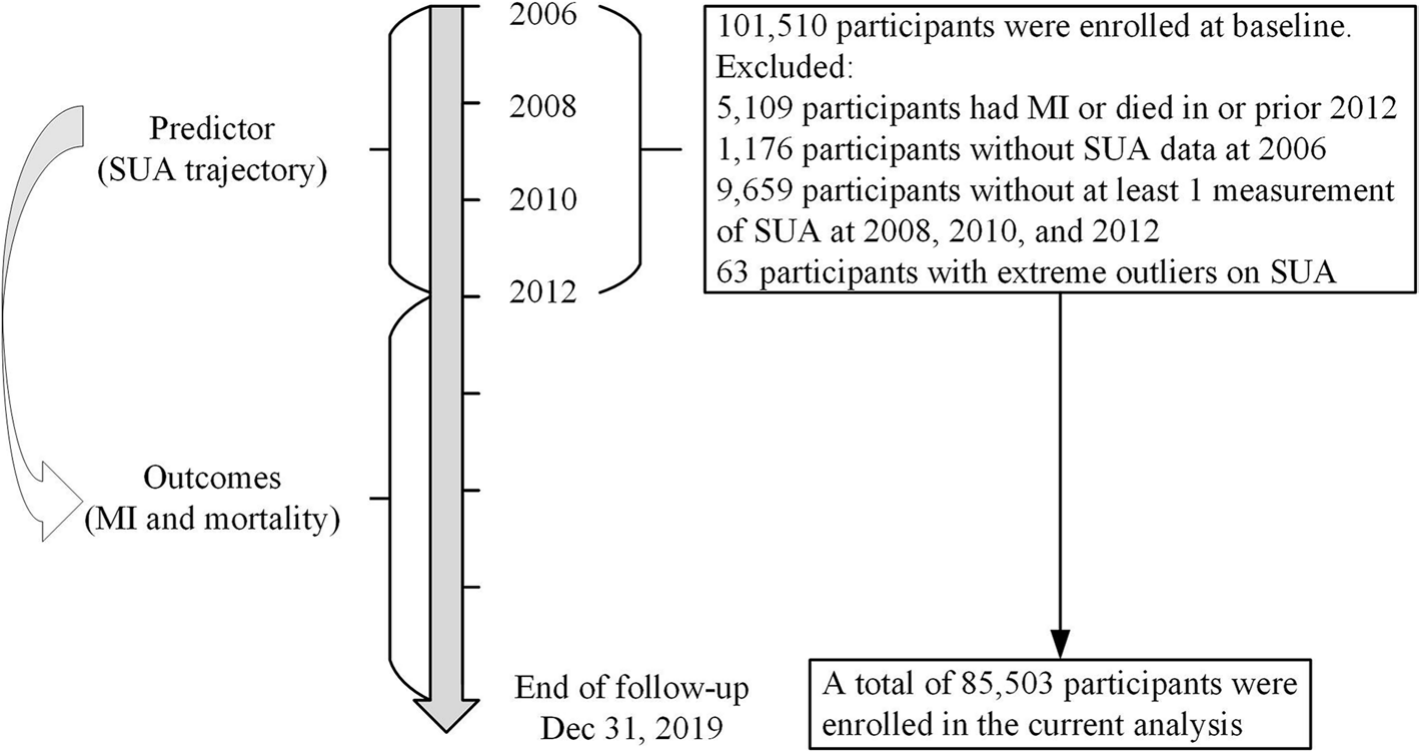
The time line and flowchart of the study. Abbreviations: MI, myocardial infarction; SUA, serum uric acid

Data were obtained from the Kailuan study, which is a community-based prospective cohort study conducted in Tangshan, China. The details of the study design and procedures have been described previously [19–21]. Since June 2006 (the baseline survey), a total of 101,510 participants (81,110 men and 20,400 women, aged 18–98 years) were enrolled from 11 hospitals in the Kailuan community and underwent questionnaire assessments, clinical examinations, and laboratory tests. Then all participants were followed up every 2 years and incidence of chronic diseases (e.g. cardiovascular disease) was recorded annually. In the present study, SUA trajectories were developed from 2006 to 2012 to predict MI and all-cause mortality risk after 2012. Participants were excluded if they had MI or died in or prior 2012 or if they did not have at least 2 measurements of SUA during 2006–2012. Following these exclusions, we included 85,530 participants in the current analysis (Fig. 1). The baseline characteristics of included participants and excluded participants due to missing data on SUA are showed in Table S1.

### Assessment of SUA

Fasting blood samples were collected in the morning after an 8- to 12-h overnight fast and transfused into vacuum tubes containing ethylene diamine tetra-acetic acid. The concentration of SUA was examined with a commercial kit (Ke Hua Biological Engineering Corporation, Shanghai, China) using an automatic biochemical analyzer (Hitachi 7600, Tokyo, Japan) according to the manufacturer’s instructions.

### Assessment of outcomes

The primary outcomes of our study were incident MI and all-cause mortality. The database of MI diagnoses was obtained from the Municipal Social Insurance Institution and Hospital Discharge Register and was updated annually during the follow-up period. We used the International Classification of Disease, 10th Revision code for the identification of potential MI (I21). Diagnosis of MI was based on a combination of chest pain symptoms, electrocardiographic signs, and cardiac enzyme levels [22]. A panel of 3 cardiologists reviewed the medical records of potential MI cases to confirm diagnosis. All-cause mortality data were gathered from provincial vital statistics offices and reviewed by physicians.

### Assessment of covariates

Demographic and clinical characteristics, including age, sex, education, income, smoking status, alcohol use, physical activity, and medical history, were collected via standardized questionnaires. Educational attainment was categorized as illiteracy or primary school, middle school, and high school or above. Income level was categorized as <800 yuan and ≥800 yuan. Smoking status was classified as never, former, or current. Alcohol consumption was calculated (in grams per day) via the frequency of intake (times per day) multiplied by the usual amount of alcoholic beverage consumed and the corresponding average ethanol content of that beverage (5.0g for 100 beer, 12.0g for 100 wine, 40.0g for hard liquor) [23, 24]. A standard drink (classified as “one severing” in the study) contained around 14.0g of ethanol.2 Based on the definition of one standard drink, we classified participants into the following categories of alcohol consumption: non-drinkers, light drinkers (women: 0–0.4 serving/day; men: 0–0.9 severing/day), moderate drinkers (women: 0.5–1.0 serving/day; men: 1.0–2.0 severing/day), and heavy drinkers (women: >1.0 serving/day; men: >2.0 severing/day) [25]. Active physical activity was defined as ≥80 minutes activity per week. Body mass index was calculated by dividing body weight (kg) by the square of height (m). Systolic blood pressure (SBP) and diastolic blood pressure (DBP) were measured 3 times with the participants in the seated position using a mercury sphygmomanometer, and the average of 3 readings was used in the analyses. All blood samples were tested using a Hitachi 747 auto-analyzer (Hitachi, Tokyo, Japan) at the central laboratory of the Kailuan Hospital. The biochemical indicators tests included fasting blood glucose (FBG), serum lipids, serum creatinine, and high sensitivity C-reactive protein (hs-CRP). Estimated glomerular filtration rate (eGFR) was calculated using the creatinine-based Chronic Kidney Disease Epidemiological Collaboration (CKD-EPI 2009) equation designed for White race [26].

Hypertension was defined as any self-reported hypertension or use of antihypertensive drug, or blood pressure ≥140/90 mm Hg. Diabetes mellitus was defined as any self-reported diabetes mellitus or use of glucose-lowering drugs, or FBG ≥7 mmol/L. Dyslipidemia was defined as any self-reported history or use of lipid-lowering drugs, or serum total cholesterol (TC) ≥5.17 mmol/L. Metabolic syndrome was defined according to updated ATP-III criteria [27].

### Statistical analyses

**Fig. 2:**
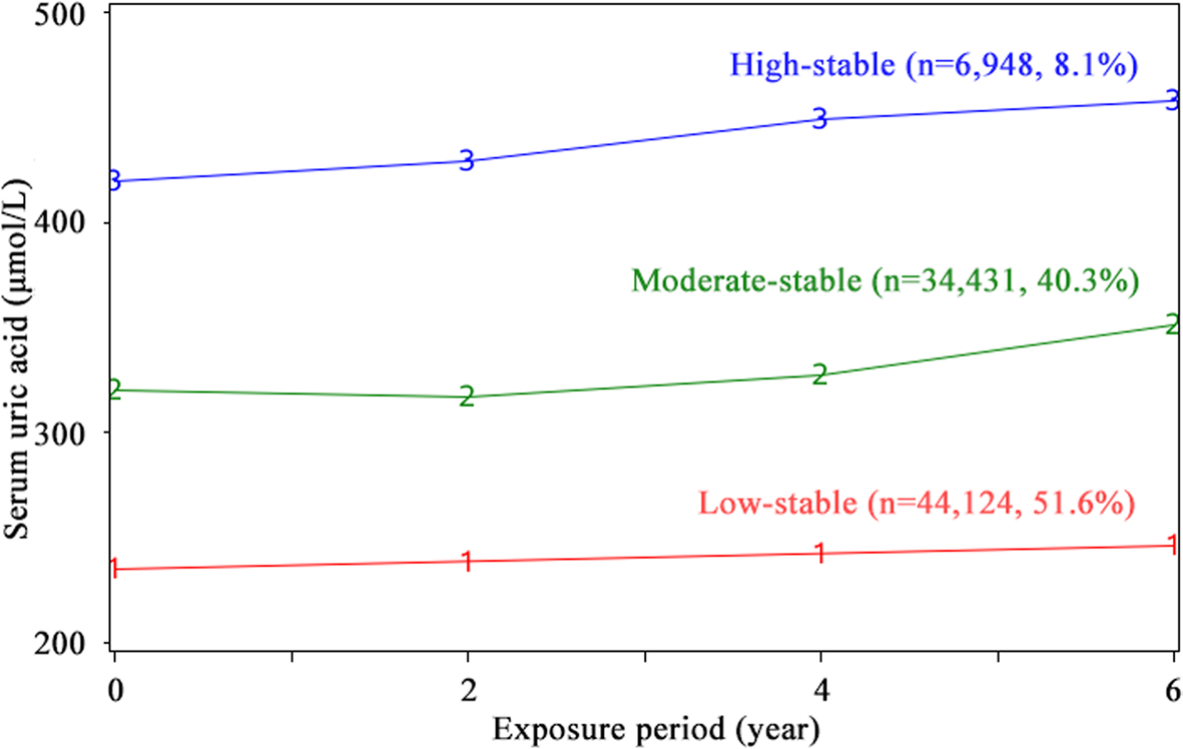
The trajectories of serum uric acid over 6 years

SUA trajectories were identified by group-based trajectory modeling using SAS PROC TRAJ [28]. This method can automatically divide the study population into classes, in such a way that participants in the same class tend to have similar trajectories of SUA change. We used a censored normal model appropriate for continuous outcomes. Model fit was assessed using the Bayesian information criterion (BIC). Initially, all SUA trajectories started with quadratic shapes and compared the BIC with the models of two, three, four and five classes. The results showed that the optimal number of trajectories was three. Next, we compared the model with different functional forms. Cubic, quadratic, and linear terms were considered and evaluated based on their significance level, starting with the highest polynomial. In our final model, we had one pattern with a linear order term and two patterns with up to quadratic order terms (Fig. 2).

Baseline characteristics were described as mean ± standard deviation (SD) for continuous variables and percentages for categorical variables. Difference in means and proportions between groups was compared using Student’s *t*-test, ANOVA, or chi-squared test, as appropriate. Person–years was computed from the date of the 2012 survey to the date of MI diagnosis, mortality, or the end of the follow-up (December 31, 2019), whichever came first. The MI and all-cause mortality probabilities were estimated by Kaplan–Meier method and compared by log–rank test.

Cox proportional hazard regression was used to examine the association between SUA trajectories from 2006 to 2012 and the risk of MI and all-cause mortality by calculating hazard ratios (HRs) and 95% confidence intervals (CIs). The models met the proportional assumption criteria according to Schoenfeld residuals and log-log inspection. Three models were constructed. Model 1 was unadjusted. Because age and sex are strong determinants of exposure and outcomes, these factors was adjusted in Model 2. We further adjusted for education, income, smoking status, drinking status, physical activity, history of hypertension, diabetes and dyslipidemia, use of antihypertensive agents, hypoglycemic agents, lipid-lowering agents, BMI, SBP, DBP, FBG, TC, eGFR, hs-CRP at baseline, and baseline SUA in model 3.

Sensitivity analyses were performed to test the robustness of our findings. First, to control the regression-to-then mean influence, we adjusted average BMI, SBP, DBP, FBG, eGFR, hs-CRP, and SUA during the exposure period. Second, to reduce the possibility of reverse causality, we conducted a lag-analysis by excluding incident MI or death, separately, with onset during the first 2 years of follow-up. Third, we adjusted for metabolic syndrome instead of its components. Fourth, we used the Fine-Gray competing risk model considering non-MI deaths as competing risk events to assess the association between SUA trajectory and MI. Fifth, we excluded participants with cardiovascular or cerebrovascular disease at baseline or during the follow-up to assess the association between SUA trajectory and all-cause, given other common diseases may have additional effects on all-cause mortality. According to baseline characteristics and previous studies [29, 30], posteriori subgroup analysis stratified by age (<60 *or* ≥60 years), sex, BMI (<25 *or* ≥25 kg/m^2^), and history of hypertension (no *or* yes) was performed to evaluate whether SUA trajectories exhibit different effect on the outcomes in special populations; interaction between stratified variables and SUA trajectories was tested using likelihood ratio.

All analyses were conducted using SAS version 9.4 (SAS Institute Inc., Cary, NC, USA). A two-sided *P*<0.05 was considered statistically significant.

## Results

### Baseline characteristics

We categorized the study population based on three observed discrete trajectories of SUA during 6-year exposure period (Fig. 2): low-stable (*n*=44,124, mean SUA ranged from 236 in 2006 to 249 μmol/L in 2010), moderate-stable (*n*=34,431, mean SUA ranged from 324 in 2006 to 354 μmol/L in 2010), and high-stable (*n*=6,984, mean SUA from 425 in 2006 to 463 μmol/L in 2010). Baseline characteristics according to SUA trajectory is presented in Table 1. Individuals with a moderate-stable and high-stable trajectories of SUA, compared with individuals with the low-stable group, were more likely to be older; be men, be educated; have a higher percentage of high income; be a current smoker; be a heavy drinker; be of lower percentage of active physical activity; have a higher prevalence of hypertension, dyslipidemia, and metabolic syndrome; take antihypertensive agents and lipid-lowering agents; and have higher levels of FBG, TC, hs-CRP, and SUA and a lower level of eGFR.

**Table 1:** Baseline characteristics of participants per trajectory of SUA

		SUA trajectory group			
Characteristics	Overall	Low-stable	Moderate-stable	High-stable	*P* value
Subjects, *n*	85,503	44,124	34,431	6948	
Age, years	50±12	50±11	50±13	51±12	<0.0001
Men, *n* (%)	66,808 (78.1)	29,615 (67.1)	30,523 (88.6)	6670 (96.0)	<0.0001
High school or above, *n* (%)	6074 (7.3)	2457 (5.7)	2785 (8.3)	832 (12.2)	<0.0001
Income≥800RMB, *n* (%)	12,172 (14.6)	5090 (11.8)	5642 (16.8)	1440 (21.2)	<0.0001
Current smoker, *n* (%)	29,102 (34.8)	11,301 (26.2)	14,416 (42.8)	3385 (49.7)	<0.0001
Alcohol use, *n* (%)					
Never or past	43,744 (66.3)	25,787 (75.1)	15,377 (58.7)	2580 (47.2)	<0.0001
Light	18,271 (27.7)	6866 (20.0)	9065 (34.6)	2340 (42.8)	
Moderate	3428 (5.2)	1513 (4.4)	1485 (5.7)	4300 (7.9)	
Heavy	370 (0.6)	117 (0.3)	168 (0.6)	85 (1.6)	
Active physical activity, *n* (%)	75,978 (91.02)	39,484 (91.6)	30,340 (90.3)	6154 (90.8)	<0.0001
Hypertension, *n* (%)	36,250 (42.4)	17,421 (39.5)	15,308 (44.5)	3521 (50.7)	<0.0001
Diabetes mellitus, *n* (%)	7411 (8.7)	4420 (10.0)	2552 (7.4)	439 (6.3)	<0.0001
Dyslipidemia, *n* (%)	29,952 (35.0)	13,180 (29.9)	13,223 (38.4)	3549 (51.1)	<0.0001
Metabolic syndrome, *n* (%)	14,717 (17.2)	6006 (13.6)	6857 (19.9)	1854 (26.7)	<0.0001
Antihypertensive agents, *n* (%)	8982 (10.5)	3039 (6.9)	4516 (13.1)	1427 (20.5)	<0.0001
Hypoglycemic agents, *n* (%)	1898 (2.2)	1047 (2.4)	709 (2.1)	142 (2.0)	0.0073
Lipid-lowering agents, *n* (%)	777 (0.9)	269 (0.6)	395 (1.2)	113 (1.6)	<0.0001
Body mass index, kg/m^2^	25±3	24±3	25±3	26±3	<0.0001
Systolic blood pressure, mmHg	130±20	128±20	131±20	134±21	<0.0001
Diastolic blood pressure, mmHg	83±11	82±11	84±11.82	86±12	<0.0001
Fasting blood glucose, mmol/L	5.4±1.6	5.5±1.7	5.3±1.3	5.4±1.5	<0.0001
Total cholesterol, mmol/L	4.9±1.1	4.8±1.2	5.1±1.0	5.2±1.1	<0.0001
eGFR, mL/min/1.73m^2^	82±25	83±23	83±27	80±25	<0.0001
hs-CRP, mg/L	2.3±6.2	2.0±5.7	2.9±6.4	3.0±7.2	<0.0001
SUA	287±82	236±53	324±58	425±74	<0.0001

Continuous variables were expressed as mean ± standard deviation and were compared with ANOVA, and categorical variables were expressed as frequency with proportion and were compared with chi-square test

*Abbreviations: eGFR* estimated glomerular filtration rate, *hs-CRP* high-sensitivity C-reactive protein; *SUA*, serum uric acid

### Association between SUA trajectories and outcomes

**Fig. 3:**
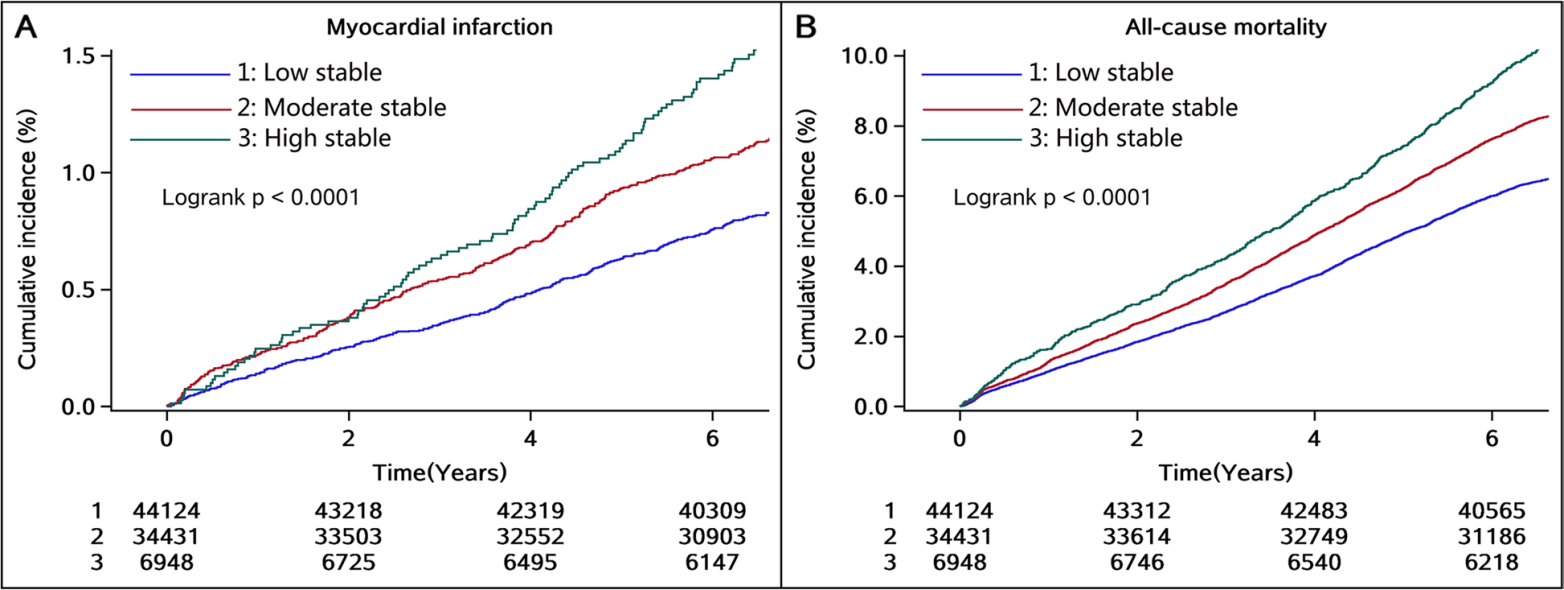
Kaplan–Meier curves of **A** myocardial infarction and **B** all-cause mortality incidence rate by serum uric acid trajectories

During a median follow-up of 6.8 years, we identified 817 (0.96%) incident MI and 6498 (7.60%) all-cause mortality. The association between SUA trajectories and risk of MI and all-cause mortality is presented in Table 2. The incidence rate of MI and all-cause mortality was increased from 1.18 (95% CI, 1.07–1.32) and 9.96 (95% CI, 9.61–10.30) per 1000 person-year in the low-stable trajectory to 2.29 (95% CI, 1.89–2.77) and 16.10 (95% CI, 14.90–17.90) per 1000 person-year in the high-stable trajectory, respectively. The Kaplan–Meier curves also showed that the high-stable trajectory had a higher risk of MI and all-cause mortality than other trajectories (log-rank test, *P*<0.0001, Fig. 3A, B).

**Table 2:** HRs (95% CIs) for incident myocardial infarction and all-cause mortality per trajectory of SUA trajectories

	SUA trajectory group			
	Low-stable	Moderate-stable	High-stable	*P* for trend
Myocardial infarction				
Cases, n (%)	342 (0.78)	372 (1.08)	103 (1.48)	<0.0001
Incidence rate^a^	1.18 (1.07–1.32)	1.66 (1.50–1.84)	2.29 (1.89–2.77)	
Model 1	Reference	1.41 (1.21–1.63)	1.94 (1.56–2.42)	<0.0001
Model 2	Reference	1.14 (0.98–1.32)	1.53 (1.22–1.91)	<0.0001
Model 3	Reference	1.10 (0.95–1.28)	1.35 (1.07–1.71)	0.0146
Sensitivity analysis^b^	Reference	1.09 (0.93–1.27)	1.33 (1.05–1.67)	0.0264
Sensitivity analysis^c^	Reference	1.06 (0.88–1.28)	1.50 (1.14–1.97)	0.0158
Sensitivity analysis^d^	Reference	1.06 (0.91–1.23)	1.33 (1.06–1.66)	0.0317
Sensitivity analysis^e^	Reference	1.10 (0.94–1.28)	1.33 (1.05–1.68)	0.0246
All-cause mortality				
Cases, *n* (%)	2886 (6.54)	2884 (8.38)	728 (10.48)	<0.0001
Incidence rate	9.96 (9.61–10.30)	12.80 (12.40–13.30)	16.10 (14.90–17.30)	
Model 1	Reference	1.29 (1.23–1.36)	1.63 (1.50–1.76)	<0.0001
Model 2	Reference	1.00 (0.95–1.05)	1.18 (1.09–1.28)	0.0040
Model 3	Reference	1.04 (0.98–1.10)	1.22 (1.12–1.33)	<0.0001
Sensitivity analysis^b^	Reference	1.04 (0.99–1.10)	1.20 (1.10–1.30)	<0.0001
Sensitivity analysis^c^	Reference	1.04 (0.98–1.11)	1.24 (1.12–1.38)	0.0003
Sensitivity analysis^d^	Reference	1.02 (0.94–1.05)	1.15 (1.06–1.26)	0.0022
Sensitivity analysis^f^	Reference	1.03 (0.97–1.10)	1.21 (1.10–1.34)	0.0010

Model 1: unadjusted

Model 2: adjusted for age, gender, and baseline SUA

Model 3: further adjusted for education, income, smoking status, alcohol use, physical activity, history of hypertension, diabetes and dyslipidemia, antihypertensive agents, hypoglycemic agents, lipid-lowering agents, body mass index, systolic blood pressure, diastolic blood pressure, fasting blood glucose, total cholesterol, estimated glomerular filtration rate, and high-sensitivity C-reactive protein

*Abbreviations: CI* Confidence interval, *HR* Hazard ratio, *SUA* Serum uric acid

^a^Incidence rate per 1000 person-years

^b^Sensitivity analysis was adjusted for average body mass index, systolic blood pressure, diastolic blood pressure, fasting blood glucose, estimated glomerular filtration rate, high-sensitivity C-reactive protein, and SUA during the exposure period and other variables at baseline in model 3

^c^Sensitivity analysis excluded incident myocardial infarction or death within the first 2 years of follow-up and adjusted for variables in model 3

^d^Sensitivity analysis was adjusted for metabolic syndrome instead of its components and other variables in model 3

^e^Sensitivity analysis was conducted using Fine-Gray competing risk model considering non-MI deaths as competing risk events and adjusted for variables in model 3

^f^Sensitivity analysis excluded participants with cardiovascular or cerebrovascular disease and adjusted for variables in model 3

Relative to the low-stable trajectory, the high-stable trajectory was associated with 35% higher risk of MI (HR, 1.35; 95% CI, 1.07–1.71) and 22% higher of all-cause mortality (HR, 1.22; 95% CI, 1.12–1.33), after being adjusted for variables in model 3. The results did not materially change by adjusting for average values of continuous covariates during the exposure period, excluding MI or mortality cases during the first 2 years of the follow-up (*n*=2057), or by using competing risk model, or by excluding cardiovascular and cerebrovascular diseases at baseline and the follow-up (*n*= 6030).

### Subgroup analysis

**Fig. 4:**
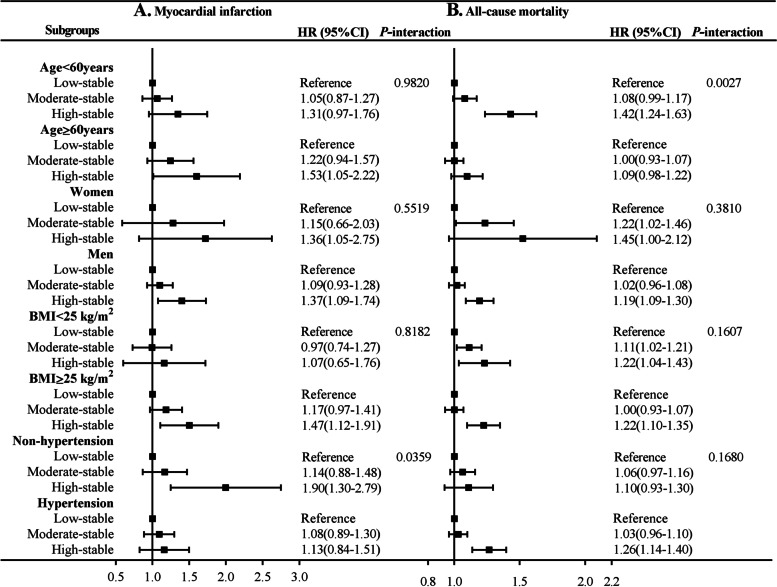
Subgroup analyses for the association with serum uric acid trajectories with risk of myocardial infarction and all-cause mortality. Abbreviations: BMI, body mass index; CI, confidence interval; HR, hazard ratio. Adjusted for age, gender, education, income, smoking status, alcohol use, physical activity, history of hypertension, diabetes and dyslipidemia, antihypertensive agents, hypoglycemic agents, lipid-lowering agents, body mass index, systolic blood pressure, diastolic blood pressure, fasting blood glucose, total cholesterol, estimated glomerular filtration rate, high-sensitivity C-reactive protein. and baseline serum uric acid other than variables for stratification

The results of subgroup analyses are shown in Fig. 4; there was significant interactions of SUA trajectories with history of hypertension and age in relation to the risk of MI (*P* for interaction=0.0359) and all-cause mortality (*P* for interaction=0.0027), respectively. The association high-stable trajectory and the risk of MI was pronounced among individuals without a history of hypertension (HR, 1.90; 95% CI, 1.30–2.79) than those with a history of hypertension (HR. 1.13; 95% CI, 0.84–1.51). Moreover, a stronger relationship between high-stable trajectory and all-cause mortality was found in young adults (aged <60 years) than elders (aged ≥60 years). The association of SUA trajectories with MI and all-cause mortality was consistent across other subgroups (*P* for interaction >0.05).

## Discussion

In this prospective cohort study, we identified 3 heterogeneous SUA trajectories, in which participants shared a similar pattern change in SUA levels during a 6-year exposure period. Participants in the high-stable group had the highest risk of MI and all-cause mortality. The associations remained robust among multiple sensitivity analyses. Posteriori subgroup analyses showed that the significant association of high-stable trajectory with MI and all-cause mortality was only observed for individuals without a history of hypertension and those aged <60 years, respectively.

The association of changes in SUA levels with MI and all-cause mortality risk remains an issue of contention [31–35]. It is worthy to note that SUA changes were measured based on SUA levels at baseline and the end of the follow-up, thereby oversimplifying the heterogeneity and complex patterns of longitudinal SUA changes. In contrast, group-based trajectory is a data-driven approach that can identify distinct clusters of individuals who are following similar trajectories and summarize the trajectory for each group over time in an easily understandable graphical depiction. With this method, our present findings identified three SUA trajectories and demonstrated that high-stable SUA during 6-year exposure period increased the risk of MI and all-cause mortality. These findings suggested that monitoring such distinct SUA trajectories may enhance the identification of at risk individuals.

Posteriori subgroup analysis found that hypertension modified the association between SUA trajectories and MI risk and subjects without hypertension exhibited a high risk of MI, while the relationship seemed insignificant in those with hypertension. This finding was supported by the Rotterdam Study that higher SUA level was significantly associated with MI risk in non-hypertensive subjects, but not in hypertensive subjects [36]. One possible explanation for the moderating effects of baseline hypertension is that the relationship between SUA and future MI risk could be mediated through developing hypertension during follow-up [37]. Therefore, the associations were weakened among participants with hypertension at baseline. This suggested that non-hypertensive individuals with high longitudinal SUA pattern warrant more particular vigilance and should be followed up more closely for the existence and development of MI risk.

In regard to all-cause mortality, when stratified by age, the association between SUA trajectories and all-cause mortality was significant among individuals aged <60 years but not in those aged ≥60 years. The reason for the discrepancy may be that young adults were more likely to have unhealthy lifestyle, which leads to levels of SUA increased rapidly which thus may cause organ damage, such as kidney disease [38]. The organ damage in young adults may be an important contributor in mortality among young adults. These finding emphasized the importance of early control of SUA during the life course.

The biological mechanisms underlying the association of SUA trajectories with MI and all-cause mortality mainly included oxidative stress, systematic inflammation, and endothelial dysfunction caused by long-term high SUA levels [39].

Furthermore, SUA trajectories have been reported to be correlated closely with cardiovascular risk factors, such as hypertension [40], diabetes [30], non-alcoholic fatty liver disease [41], and chronic kidney disease [42], which can contribute to the development of MI and reduce the longevity of the affected individuals.

### Strengths and limitations

The strengths of the present study include its large sample size, prospective longitudinal design, long follow-up period, repeated measurements of SUA levels, and the applied SUA longitudinal trajectories to estimate the risk of MI and all-cause mortality. However, our study also has several limitations. First, a history of gout and the mediations for hyperuricemia or gout were not recorded in our study. Second, information on fructose-soft drinks was not available in our database, which may significantly increase SUA levels [43, 44]. However, since our study population were mainly middle-age or elderly, the proportion of participants with fructose-soft drinks may be low and thus may not have a substantial influence on the results. Third, we did not collect information on specific causes of death, while we performed sensitivity analyses by excluding those whose deaths were from cardiovascular and cerebrovascular diseases at baseline and during the follow up.

## Conclusions

In conclusion, our study found that high-stable SUA trajectories were associated with an increased risk of MI and all-cause mortality. Furthermore, subgroup analysis showed the association of SUA trajectories with MI and all-cause mortality was higher in non-hypertensive subjects and young adults, respectively. These findings suggested that monitoring long-term SUA patterns may contribute additional information for identifying subjects at higher risk of MI and all-cause mortality, and highlight the importance of maintaining normal SUA during the life course.

## Supplementary Information

**Additional file 1: Table S1.** Baseline characteristics of included and excluded participants due to missing data on SUA.

## Data Availability

The data generated by our research could be made available upon request to the corresponding authors.
