# Supplementary material for: High serum uric acid trajectories are associated with risk of myocardial infarction and all-cause mortality in general Chinese population
Source: Arthritis Res Ther. 2022 Jun 21;24:149. doi: 10.1186/s13075-022-02812-y (PMC9210742; doi:10.1186/s13075-022-02812-y)
Supplement: Supplementary file 1 — Additional file 1: Table S1. Baseline characteristics of included and excluded participants due to missing data on SUA. [file 13075_2022_2812_MOESM1_ESM.docx]

**SUPPLEMENTAL MATERIALS**

Table S1. Baseline characteristics of included and excluded participants due to missing data on SUA

|  | Excluded | Included | *P* value |
| --- | --- | --- | --- |
| Subjects, n | 10898 | 85503 |  |
| Age, years | 57.85±13.83 | 50.45±11.94 | <0.0001 |
| Men, n (%) | 9576 (87.87) | 66808 (78.14) | <0.0001 |
| High school or above, n (%) | 550 (6.04) | 6074 (7.26) | <0.0001 |
| Income≥800RMB, n (%) | 1192 (13.08) | 12172 (14.56) | 0.0001 |
| Current smoker, n (%) | 3053 (31.27) | 29102 (34.77) | <0.0001 |
| Current alcohol, n (%) | 3130 (32.07) | 32111 (38.35) | <0.0001 |
| Active physical activity, n (%) | 8493 (93.29) | 75978 (91.02) | <0.0001 |
| Hypertension, n (%) | 5145 (47.21) | 36250 (42.40) | <0.0001 |
| Diabetes mellitus, n (%) | 1135 (10.41) | 7411 (8.67) | <0.0001 |
| Dyslipidemia, n (%) | 4382 (40.21) | 29952 (35.03) | <0.0001 |
| Antihypertensive agents, n (%) | 1196 (10.98) | 8982 (10.50) | 0.1322 |
| Hypoglycemic agents, n (%) | 255 (2.34) | 1898 (2.22) | 0.4235 |
| Lipid-lowering agents, n (%) | 81 (0.74) | 777 (0.91) | 0.0834 |
| Body mass index, kg/m^2^ | 24.87±3.61 | 25.07±3.47 | <0.0001 |
| SBP, mmHg | 134.73±22.28 | 129.99±20.53 | <0.0001 |
| DBP, mmHg | 84.00±12.12 | 83.28±11.65 | <0.0001 |
| FBG, mmol/L | 5.62±1.93 | 5.44±1.60 | <0.0001 |
| eGFR, mL/min/1.73m^2^ | 4.94±1.16 | 4.95±1.14 | 0.6960 |
| Total cholesterol, mmol/L | 1.69±1.42 | 1.67±1.37 | 0.4174 |
| hs-CRP, mg/L | 2.67±7.66 | 2.30±6.17 | <0.0001 |

Abbreviations: DBP, diastolic blood pressure; eGFR, estimated glomerular filtration rate; FBG, fasting blood glucose; HDL, high density lipoprotein; hs-CRP, high-sensitivity C-reactive protein; LDL, low density lipoprotein; SBP, systolic blood pressure.
